# Impact of T-AMYLO Risk Score and Red Flag Findings on Cardiovascular Outcomes in Patients with Cardiac Conduction Defects Treated with Intracardiac Device Implantation

**DOI:** 10.3390/jcdd12110424

**Published:** 2025-10-26

**Authors:** Hidayet Ozan Arabaci, Sukru Arslan, Cem Kurt, Pelinsu Hunkar, Fatih Ozkan, Muhammet Heja Gecit, Seyma Arslan, Mustafa Yildiz

**Affiliations:** 1 Department of Cardiology, Gaziosmanpasa Training and Research Hospital, Istanbul 34255, Türkiye; 2 Institute of Cardiology, Istanbul University-Cerrahpasa, Istanbul 34098, Türkiye; sukru.arslan@iuc.edu.tr (S.A.); cem-kal@hotmail.com (C.K.); hunkarpelinsu@gmail.com (P.H.); gecitheja@gmail.com (M.H.G.); mustafayilldiz@yahoo.com (M.Y.); 3 Department of Cardiology, Istanbul Training and Research Hospital, Istanbul 34098, Türkiye; fatihozkan.fo@gmail.com; 4 Republic of Türkiye Ministry of Health, General Directorate of Public Health, Ankara 06800, Türkiye; sheyma87@gmail.com

**Keywords:** cardiac amyloidosis, red flags, T-AMYLO score, cardiac conduction defects, cardiac implantable electronic devices

## Abstract

Background: Cardiac amyloidosis is more common than previously thought with an incidence of up to 15% in aortic stenosis and heart failure with preserved ejection fraction. Pacemaker need in these patients ranges from 9.5% to 20%; however, its prevalence and clinical relevance in patients with unexplained cardiac conduction defects remain unclear. Methods: This retrospective, single-center cohort study evaluated 1107 patients who underwent intracardiac device implantation for unexplained cardiac conduction defects between 2015 and 2024. Patients with secondary conduction defects or known cardiomyopathy were excluded. The prognostic value of the T-AMYLO score and associated red flag findings were assessed in relation to the composite primary endpoint: all-cause mortality, non-fatal myocardial infarction, and non-fatal stroke. Results: Over a median of 58 months for follow-up, 460 patients experienced a primary event, including 346 deaths. Higher event rates were observed in older males, those with atrioventricular block, and patients receiving single-lead ventricular devices. T-AMYLO score and the presence of red flag findings, particularly aortic valve disease, AV block, peripheral neuropathy, low voltages and increased septal thickness were significantly associated with adverse outcomes. Multivariate Cox regression identified elevated T-AMYLO score (HR: 1.06, *p* = 0.012), aortic valve disease (HR: 1.29, *p* = 0.016), and AV block (HR: 1.43, *p* = 0.009) as independent predictors of mortality. Survival analyses confirmed a stepwise decline in prognosis with an increasing T-AMYLO risk group and red flag burden (*p* < 0.001). Conclusion: These findings highlight the importance of incorporating T-AMYLO scoring and red flags assessment in patients with conduction defects to improve early detection of cardiac amyloidosis and guide risk stratification for outcomes.

## 1. Introduction

Cardiac amyloidosis (CA) is an infiltrative and progressive disorder characterized by the extracellular deposition of misfolded, insoluble precursor proteins within the myocardium [1]. It may result from the accumulation of misfolded monoclonal light chains due to plasma cell dyscrasias (AL-CA) or from misfolded transthyretin proteins (ATTR-CA) [2]. ATTR-CA, contrary to previous beliefs of rarity, has become increasingly recognized in recent years due to heightened clinical awareness, identification of red flag features, and the development of risk scoring systems that facilitate earlier diagnosis. Moreover, the advent of disease-modifying therapies such as tafamidis has been associated with improved survival outcomes [3]. Recent studies have identified ATTR-CA in approximately 15% of patients with heart failure with preserved ejection fraction (HFpEF) and in 13% of those undergoing transcatheter aortic valve implantation (TAVI) [4,5]. Among the key red flag findings, cardiac conduction disorders, particularly atrioventricular (AV) blocks, are reported in 9.5% to 20% of patients diagnosed with ATTR-CA [6]. However, the prevalence of CA among patients receiving intracardiac device implantation due to conduction abnormalities in the general population remains unclear.

Diagnostic scoring systems developed for ATTR-CA have been primarily validated in populations with HFpEF and aortic valve disease [7,8]. Among them, the T-AMYLO risk score has emerged as a promising tool due to its ease of clinical application and high diagnostic performance in these groups [7]. Nevertheless, the applicability and validation of such risk models remain limited in patients with cardiac conduction disorders undergoing intracardiac device implantation, and their prognostic implications on cardiovascular outcomes have not been fully established. In this study, we aimed to evaluate the impact of the T-AMYLO score and predefined red flag findings for cardiac amyloidosis [9] on adverse cardiovascular outcomes, particularly all-cause mortality, in patients receiving intracardiac devices for unexplained cardiac conduction abnormalities.

## 2. Materials and Methods

Study population: This retrospective, cross-sectional, and observational study included patients who underwent intracardiac device (CIED) implantation at the Arrhythmia and Electrophysiology Clinic of our university’s Institute of Cardiology between January 2015 and June 2024. During this period, a total of 3052 patients received a CIED. Patients aged 18 years or older, who underwent device implantation due to a cardiac conduction disorder and had accessible clinical data from hospital and national electronic medical records, were considered eligible for inclusion.

Cardiac conduction disorders requiring device implantation were categorized into four primary indications, based on guidelines from the European Society of Cardiology and the American Heart Association [10,11]. These indications included: atrioventricular (AV) blocks, non-AV block conduction disorders, sick sinus syndrome (SSS) or symptomatic sinus bradycardia, and atrial fibrillation (AF) with a slow ventricular rate. The AV block category comprised complete AV block, high-grade AV block, and 2:1 AV block. Non-AV block conduction disorders included bifascicular block or trifascicular block in the presence of symptomatic bradycardia. Slow ventricular rate in AF was defined as a baseline rhythm of permanent AF accompanied by symptomatic bradyarrhythmia with a heart rate < 50 bpm or a symptomatic pause > 3 s [10,11].

**Figure 1:**
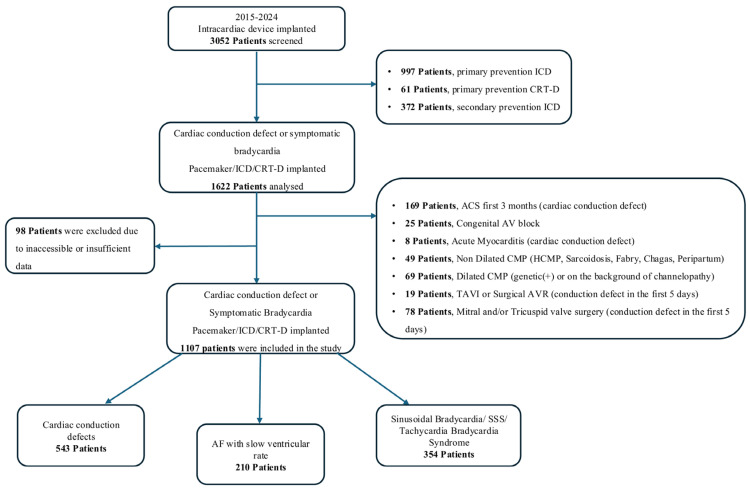
A Flowchart of The Study. ACS: acute coronary syndromes, AF: atrial fibrillation, AV: atrioventricular, AVR: aortic valve replacement, CMP: cardiomyopathy CRT-D: cardiac resynchronisation therapy with defibrillator, HCMP: hypertrophic cardiomyopathy, ICD: implantable cardioverter-defibrillator, SSS: sick sinus syndrome, TAVI: transcatheter aortic valve implantation.

From the 3052 patients reviewed, those who received an implantable cardioverter-defibrillator (ICD) for primary or secondary prevention or a cardiac resynchronization therapy (CRT-D) for primary prevention were excluded, resulting in 1622 eligible patients. Further exclusions were applied to patients with clearly defined conduction defect etiologies: those with post-acute coronary syndrome conduction disorders within the first 3 months, congenital AV block, myocarditis-related conduction abnormalities, genetically confirmed hypertrophic or dilated cardiomyopathy, infiltrative or metabolic cardiomyopathies such as sarcoidosis or Fabry disease, as well as patients who developed conduction disorders following surgical or transcatheter aortic valve interventions, or mitral/tricuspid valve surgery. Additionally, patients with incomplete or inaccessible medical records were excluded. After applying all criteria, a total of 1107 patients were included in the final analysis. The study flowchart is presented in Figure 1.

Initial demographic characteristics, laboratory parameters, electrocardiographic (ECG) and echocardiographic (ECHO) findings, physical examination data, and current medications were retrieved from institutional and national electronic health records. Coronary artery disease (CAD) was defined as the presence of ≥50% stenosis on invasive coronary angiography or a history of percutaneous coronary intervention or coronary artery bypass graft surgery. Chronic kidney disease (CKD) was defined as a glomerular filtration rate (GFR) < 60 mL/min. Aortic valve disease was categorized as mild-to-moderate (regurgitation and/or stenosis), severe (advanced regurgitation and/or stenosis), or prior surgical or transcatheter aortic valve replacement (SAVR or TAVI). In cases of inconsistent or missing data, information was verified via phone or email communication with patients or caregivers. The study was conducted in accordance with the Declaration of Helsinki and was approved by the Institutional Review Board of Istanbul University-Cerrahpasa (E-74555795-050.04-1036119, 8 May 2024).

Electrocardiographic (ECG) parameters analyzed included baseline rhythm, PR interval, QRS duration, QTc interval (calculated using Bazett’s formula), presence of left or right bundle branch block (LBBB/RBBB), fragmented QRS, low voltage criteria, and AV block. All ECG measurements were obtained from pre-implantation baseline ECGs and expressed in milliseconds. Low voltage was defined as QRS amplitude <5 mm in all limb leads or <10 mm in all precordial leads [12]. Echocardiographic (ECHO) parameters were retrospectively assessed from transthoracic ECHO images and reports. Parameters included interventricular septal thickness (IVS), posterior wall thickness (PW), left ventricular end-diastolic diameter (LVEDd), left ventricular ejection fraction (LVEF), left atrial diameter (measured posterior–anterior), left ventricular diastolic dysfunction (LVDD), tricuspid annular plane systolic excursion (TAPSE), and systolic pulmonary artery pressure (sPAP). All measurements were recorded in millimeters.

T-AMYLO risk score calculation: The T-AMYLO score was calculated for each patient using clinical, ECG, and ECHO data via the official website (https://www.t-amylo.com) [7]. Score calculation requires an IVS thickness ≥12 mm; patients below this threshold were not scored. The T-AMYLO score consists of five parameters: age, male sex, IVS thickness, history of carpal tunnel syndrome (CTS), and presence of low voltage on ECG. The tool provides a numerical probability (%) for transthyretin-type cardiac amyloidosis (ATTR-CA). Risk <20% was classified as low, 20–74.9% as intermediate, and ≥75% as high. Based on these thresholds, patients were stratified into four groups: undetermined, low, intermediate, and high-risk, and analyzed for primary endpoints and all-cause mortality.

Red flag criteria for cardiac amyloidosis: Red flag findings were defined according to the European Society of Cardiology guidelines [9]. These included: age > 65 years, IVS > 12 mm, presence of aortic valve disease, HFpEF, LVDD, CTS, peripheral neuropathy, autonomic dysfunction, AV block on ECG, low voltage and/or pseudo-infarct pattern on ECG. Autonomic dysfunction was defined as the presence of constipation/diarrhea episodes, incontinence, orthostatic tachycardia or hypotension, or inappropriate tachycardia. CTS was defined as any sensory or motor neuropathy symptoms due to median nerve compression at the wrist or a history of surgery for CTS. Peripheral neuropathy was defined as entrapment-related neuropathies originating outside the wrist, including sensory or motor symptoms caused by median nerve compression at the proximal forearm or shoulder level, as well as femoral and/or sciatic nerve involvement due to spinal canal stenosis or herniation in the lumbosacral vertebral region. Aortic valve disease was considered a red flag if any degree of stenosis or regurgitation with leaflet thickening or degeneration was observed on ECHO. AV block was defined as a red flag in cases of bifascicular/trifascicular block with symptomatic bradycardia, Mobitz type II or higher-degree AV block, complete AV block, or slow ventricular rate-AF requiring CIED. LVDD was considered a red flag if ECHO showed left atrial enlargement with sPAP >28 mmHg and impaired LV diastolic function parameters.

Study endpoints and follow-up: Primary endpoints included all-cause mortality, non-fatal myocardial infarction, and non-fatal stroke. Follow-up duration was defined as the time from device implantation to June 2024 for patients meeting all inclusion criteria.

Statistical analysis: All statistical analyses were performed using SPSS Statistics version 24.0 (SPSS Inc., Chicago, Illinois). Continuous variables were tested for normality and analyzed using Student’s *t*-test or Mann–Whitney U test as appropriate. Categorical variables were compared using Pearson’s chi-square test. Continuous data were presented as mean ± standard deviation or median (min–max), and categorical data as frequency (%). Independent predictors of the primary endpoint and all-cause mortality were identified using multivariate Cox regression, including variables with *p* < 0.10 in univariate analyses or clinical relevance. The generalizability of the final model to the overall patient population was assessed using binary logistic regression, and the explanatory power of the model was reported as Nagelkerke R^2^. Long-term outcomes by T-AMYLO risk group and total red flag burden were assessed using Kaplan–Meier survival analysis. Predictive accuracy of T-AMYLO score risk groups and red flag burden for both outcomes were evaluated using Receiver Operating Characteristic (ROC) curves. A *p*-value < 0.05 was considered statistically significant.

## 3. Results

A total of 1107 patients were included in the study; 51.6% were male, and the median age was 75.4 years. Regarding comorbidities, diabetes mellitus was present in 39.2%, hypertension in 83.3%, and coronary artery disease in 36.6% of patients. AF was observed in 541 patients (49.1%), and chronic kidney disease in 323 patients (29.2%). Among conduction disorders, AV block was observed in 39.9% (442 patients), non-AV block bundle branch defects in 9.1% (101 patients), sick sinus syndrome or symptomatic sinus bradycardia in 32.0% (354 patients), and AF with slow ventricular rate in 19.0% (210 patients). The primary endpoint was observed in 470 patients (42.5%). When analyzed by individual subcomponents, all-cause mortality occurred in 346 patients (31.3%), non-fatal myocardial infarction in 155 patients (14.0%), and non-fatal cerebrovascular events in 65 patients (5.9%).

Table 1 presents detailed clinical and demographic characteristics according to the occurrence of primary endpoints and all-cause mortality. A total of 470 patients experienced a primary endpoint, with 346 of these being all-cause mortality. Patients who experienced a primary event were significantly older (median age 82 vs. 74, *p* < 0.001) and more often male (58.7%, *p* < 0.001). Primary events were more common among patients with AV block (35.5% vs. 46.0%, *p* < 0.001) and slow-ventricular-rate AF, while they were less frequent in those with sick sinus syndrome or symptomatic sinus bradycardia (39.1% vs. 22.3%, *p* < 0.001). Primary endpoints were also more frequent in patients implanted with VR-pacemakers and VR-ICDs (20.1% vs. 29.6%; 5.3% vs. 13.2%, respectively; *p* < 0.001). Among comorbidities, the presence of diabetes, hypertension, CKD, CAD, cerebrovascular events, and AF were all significantly associated with primary endpoints (*p* < 0.001 for each, except AF *p* = 0.002).

Patients who experienced all-cause mortality were older (median age 84 vs. 74, *p* < 0.001) and predominantly male (58.1%, *p* = 0.003). The highest mortality rates were observed among patients with AV block (35.5% vs. 49.7%, *p* < 0.001) and slow-ventricular-rate AF (16.4% vs. 24.6%, *p* < 0.001). Regarding device type, mortality was significantly higher in patients with VR-pacemakers (19.2% vs. 35.0%) and VR-ICDs (6.4% vs. 13.6%) (*p* < 0.001 for both). The presence of diabetes, hypertension, CKD, cerebrovascular disease, CAD, and AF was also significantly associated with increased all-cause mortality (*p* < 0.001 for all, except CAD *p* = 0.001). Among patients with aortic valve disease, those who had undergone surgical or transcatheter aortic valve replacement (SAVR/TAVI) had significantly higher mortality (5.9% vs. 11.6%, *p* < 0.001). Use of medical therapies was also more frequent in patients who died.

Electrocardiographic and echocardiographic parameters in relation to primary endpoints and all-cause mortality are presented in Table 2. Adverse outcomes were more frequently observed in patients with a pacemaker rhythm on ECG (32.2% vs. 46.6%, *p* < 0.001). Additionally, PR interval, QTc duration, and QRS duration were significantly longer in patients who experienced primary endpoints (*p* = 0.048, *p* < 0.001, and *p* = 0.025, respectively). The presence of complete LBBB was associated with a higher incidence of primary endpoints (*p* < 0.001). Echocardiographically, patients with primary endpoints had lower LVEF and greater LA diameter, LVEDd and IVS thickness (*p* = 0.001, *p* < 0.001, *p* < 0.001, and *p* = 0.003, respectively).

Similarly, patients who experienced all-cause mortality were more likely to exhibit a pacemaker rhythm (29.0% vs. 51.7%, *p* < 0.001). PR interval, QTc, and QRS durations were also significantly prolonged in these patients (*p* = 0.007, *p* < 0.001, and *p* < 0.001, respectively). The presence of complete LBBB was strongly associated with increased mortality (43.2% vs. 69.6%, *p* < 0.001), as was complete AV block (*p* < 0.001). Low voltage findings on ECG were also more prevalent among patients who died (*p* = 0.003). On echocardiography, patients experienced all-cause mortality had significantly lower LVEF and larger LA diameter and LVEDd (*p* = 0.002, *p* < 0.001, and *p* < 0.001, respectively). Likewise, IVS thickness was also greater in this group (*p* = 0.013).

The distribution of biochemical and laboratory parameters according to the occurrence of primary endpoints and all-cause mortality is presented in Table 3. Patients who experienced a primary endpoint had significantly lower levels of Hb, Hct and PLT (*p* < 0.001, *p* < 0.001, and *p* < 0.001, respectively). In the same group, neutrophil and monocyte counts were higher (4.3 vs. 4.7, *p* < 0.001; 0.57 vs. 0.60, *p* = 0.010, respectively). Among biochemical markers, serum creatinine levels were significantly higher (0.9 vs. 1.1 mg/dL, *p* < 0.001), while estimated GFR was significantly lower (69 vs. 54 mL/min, *p* < 0.001) in patients with a primary endpoint. Additionally, levels of cardiac biomarkers such as troponin T (*p* < 0.001) and NT-proBNP (*p* < 0.001), as well as the inflammatory marker C-reactive protein (CRP) (*p* < 0.001), were significantly elevated in this group.

Similarly, patients who experienced all-cause mortality also had lower Hb, Hct, and PLT values (*p* < 0.001 for all). Neutrophil (4.3 vs. 4.9, *p* < 0.001) and monocyte (0.56 vs. 0.60, *p* < 0.001) counts were significantly higher in patients who died. Elevated levels of troponin T and NT-proBNP (*p* < 0.001 for both) were strongly associated with mortality. Likewise, CRP levels were significantly higher among patients who experienced all-cause mortality (*p* < 0.001).

The relationship between the T-AMYLO score and risk categories, as well as the presence of cardiac amyloidosis-associated red flag findings with the development of primary endpoints and all-cause mortality, is detailed in Table 4. The cumulative number of red flag findings present in each patient was calculated as the total red flag count, with a median value of 5 (range: 0–9) in the overall study population.

Patients who experienced a primary endpoint had significantly higher T-AMYLO scores (1.0 vs. 2.9, *p* < 0.001). The incidence of adverse outcomes was markedly higher among those categorized in the moderate and high-risk T-AMYLO groups (11.5% vs. 21.7%; 4.7% vs. 8.3%, respectively; *p* < 0.001). Among the individual red flag indicators, the presence of aortic valve disease, HF with LVEF > 40%, and age over 65 years was significantly more common in patients with primary endpoints (*p* < 0.001, *p* = 0.006, and *p* < 0.001, respectively). In addition, peripheral neuropathy, AV block, and IVS thickness > 12 mm were also significantly associated with primary outcome occurrence (*p* < 0.001, *p* < 0.001, and *p* = 0.008, respectively). The total red flag count was also significantly higher in this group (median: 4.5 vs. 5.5, *p* < 0.001).

Similarly, patients who experienced all-cause mortality had significantly higher T-AMYLO scores (0.2 vs. 4.8, *p* < 0.001). Mortality rates were substantially increased in patients classified as moderate and high-risk according to the T-AMYLO score (12.2% vs. 23.7%; 4.7% vs. 9.5%, respectively; *p* < 0.001). Red flag indicators such as aortic valve disease, HF with LVEF > 40%, and age over 65 years were significantly more prevalent among those who died (*p* < 0.001, *p* = 0.004, and *p* < 0.001, respectively). Peripheral neuropathy (22.5% vs. 8.3%, *p* < 0.001) and AV block (84.1% vs. 65.0%, *p* < 0.001) emerged as strong predictors of mortality. The combination of LVH with low voltage on ECG (14.1% vs. 18.8%, *p* = 0.044) and IVS > 12 mm (45.3% vs. 52.9%, *p* = 0.020) were also significantly more frequent in patients with all-cause mortality. Taken together, these findings demonstrate that the total red flag count was notably higher among patients who died (median: 5.0 vs. 6.0, *p* < 0.001).

Table 5 presents the Multivariate Cox Regression model developed to identify independent predictors of all-cause mortality. According to the analysis, decreased estimated GFR (HR: 0.98, *p* < 0.001), elevated NT-proBNP (HR: 1.01, *p* = 0.001), and elevated CRP levels (HR: 1.01, *p* < 0.001) were found to be independent predictors of mortality. Among ECG parameters, the presence of complete LBBB was significantly associated with increased mortality risk (HR: 1.43, *p* = 0.002). From the predefined red flag findings, aortic valve disease (HR: 1.29, *p* = 0.030), HF with LVEF > 40% (HR: 1.42, *p* = 0.037), and peripheral neuropathy (HR: 1.52, *p* = 0.002) were independently associated with all-cause mortality. Additionally, the presence of AV block emerged as an independent predictor of death (HR: 1.48, *p* = 0.013) and also a higher T-AMYLO risk score was also identified as a significant predictor of mortality (HR: 1.00, *p* = 0.002). The generalizability of the Multivariate Cox Regression model to the broader study population was confirmed through binary logistic regression, with a calculated Nagelkerke R^2^ value of 0.412.

**Figure 2:**
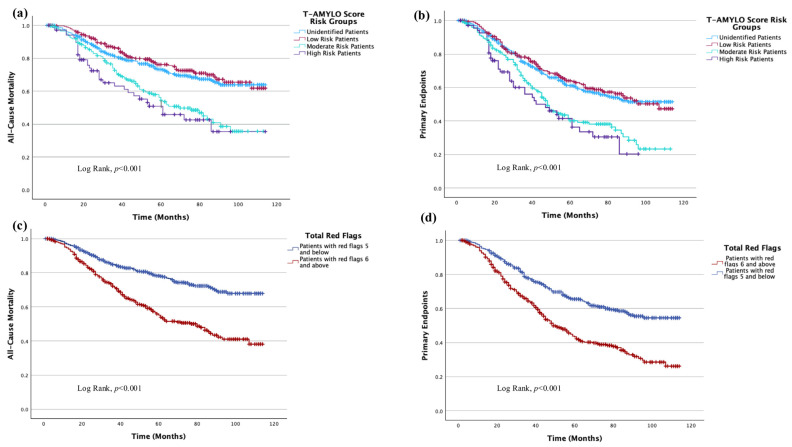
(**a**–**d**). Kaplan–Meier Curves Demonstrating the Impact of T-AMYLO Risk Score and Total Red Flag Findings on Survival Outcomes. (**a**) Presents the association between *T-AMYLO* score risk groups and the development of all-cause mortality. (**b**) Presents the association between *T-AMYLO* score risk groups and the occurrence of primary endpoints. (**c**) Presents the association between the total number of red-flag findings and the development of all-cause mortality. (**d**) Presents the association between the total number of red-flag findings and the occurrence of primary endpoints.

The prognostic impact of the T-AMYLO risk score and the total number of red flag findings on survival outcomes is illustrated in Figure 2. Figure 2a demonstrates a significant decline in survival as the T-AMYLO risk category increases (*p* < 0.001). Similarly, in Figure 2b, an increase in the T-AMYLO risk group is associated with a significantly higher incidence of primary endpoints, particularly among patients classified as moderate and high risk, along with a marked reduction in overall survival (*p* < 0.001).

In Figure 2c,d, patients with a total red flag count of ≥6 exhibited significantly higher rates of both primary endpoints and all-cause mortality, accompanied by a pronounced decrease in survival (*p* < 0.001 and *p* < 0.001, respectively).

**Figure 3:**
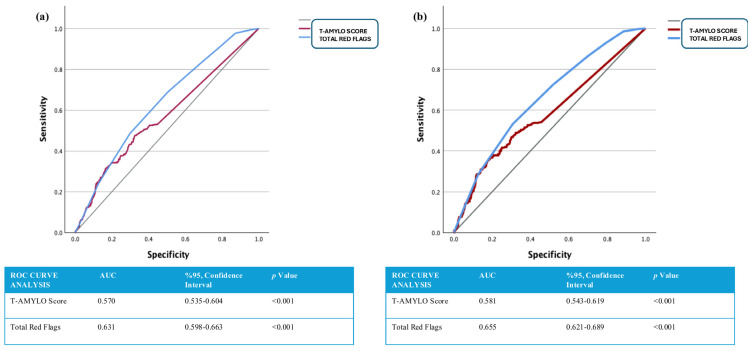
(**a**,**b**). ROC Curve Analyses Demonstrating the Impact of T-AMYLO Risk Score and Total Red Flag Findings on Primary Endpoints and All-Cause Mortality. (**a**) Presents the predictive value of the total number of red-flag findings and the *T-AMYLO* risk score for the occurrence of primary endpoints, as assessed by ROC curve analysis. (**b**) Presents the predictive value of the total number of red-flag findings and the *T-AMYLO* risk score for the development of all-cause mortality, as assessed by ROC curve analysis.

The ROC curve analysis illustrating the predictive performance of the T-AMYLO risk score and the total number of red flag findings for adverse outcomes and mortality is presented in Figure 3. In Figure 3a, the total red flag count demonstrated superior predictive value for primary endpoint occurrence compared to the T-AMYLO risk score (T-AMYLO score: AUC = 0.570, 95% CI: 0.535–0.604, *p* < 0.001; total red flag count: AUC = 0.631, 95% CI: 0.598–0.663, *p* < 0.001). Similarly, Figure 3b shows that the total red flag count outperformed the T-AMYLO score in predicting all-cause mortality (T-AMYLO score: AUC = 0.581, 95% CI: 0.543–0.619, *p* < 0.001; total red flag count: AUC = 0.655, 95% CI: 0.621–0.689, *p* < 0.001).

## 4. Discussion

In our study, a higher T-AMYLO score and the presence of red flag findings were found to be significantly associated with increased rates of both all-cause mortality and primary outcomes in patients who underwent intracardiac device implantation due to cardiac conduction disorders. Furthermore, both the T-AMYLO score and the total number of red flag findings were identified as independent predictors of mortality in the Multivariate Cox Regression model. An increase in the T-AMYLO score and a total red flag count of six or more were also significantly associated with reduced survival in Kaplan–Meier analyses. Finally, ROC curve analysis revealed that a higher number of red flag findings had superior predictive accuracy for all-cause mortality compared to the T-AMYLO score.

Our study also demonstrated that patients who underwent device implantation due to high-grade AV block or atrial fibrillation with a slow ventricular rate were significantly overrepresented among those who experienced both primary endpoints and all-cause mortality. These findings are consistent with previous literature and may be attributed to the deleterious effects of right ventricular pacing–induced electrical and mechanical dyssynchrony, which can ultimately lead to pacing-induced cardiomyopathy [13,14,15]. In addition, the proportion of patients receiving VR-pacemakers or VR-ICDs was significantly higher in those who experienced adverse outcomes. This association is likely due to both the increased burden of ventricular pacing and the higher prevalence of permanent atrial fibrillation and comorbidities in these patients [16,17].

In the original T-AMYLO study by Arana-Achaga et al., permanent pacemaker implantation was reported in 17.6% of patients diagnosed with ATTR-CA [7]. In a prospective study conducted in Türkiye by Yalvaç HE et al., which included patients with HFpEF and at least three red flag features, ATTR-CA was identified in 19 patients (11.3%). Notably, PR interval prolongation was found to be significantly higher in the ECGs of patients diagnosed with CA, while the prevalence of AV block, although increased, did not reach statistical significance [18]. The authors identified age over 65, pseudo-infarct pattern on ECG, low QRS voltage, and reduced left ventricular global longitudinal strain (LV-GLS) as independent predictors of ATTR-CA. While peripheral neuropathy and AV conduction abnormalities were commonly observed among ATTR-CA patients, CTS was reported in only two cases [18]. Similarly, in a case series reported in a letter to the editor by Yılmaz I et al., all patients were diagnosed with atrial fibrillation, yet none had CTS [19]. The authors emphasized the potential geographic variability of red flag features and advocated for population-specific red flag profiling.

A recent study by Shingu M. et al. also identified cardiac conduction disorders as independent predictors of ATTR-CA. Additional predictors included IVS thickness > 12 mm, the presence of entrapment-related peripheral neuropathy, elevated troponin levels and the absence of hypertension [20]. The reported prevalence of ATTR-CA in elderly patients undergoing device implantation for high-grade AV block varies widely in the literature. In a Spanish cohort, López-Sainz Á. et al. diagnosed ATTR-CA in only 2 out of 111 pacemaker recipients [21]. Similarly, in a study by Cannie et al., only 3 of 355 pacemaker-implanted patients were found to have ATTR-CA [22]. However, in a study by Aaseth E. et al., 57 patients with IVS thickness > 12 mm who had undergone CIED implantation were evaluated, and ATTR-CA was diagnosed in 11 of them. Notably, 80% of these patients had received the device due to high-grade AV block. These findings suggest that in this specific phenotype, the prevalence of ATTR-CA can reach as high as 19% [23]. Cardiac conduction disorders are frequently observed in patients with established ATTR-CA. In a study by Saturi et al., the prevalence of CIEDs was 10% at diagnosis and increased to 11.6% over a 16-month follow-up [6]. Donnellan et al. similarly reported that 9.5% of ATTR-CA patients had a pacemaker at the time of diagnosis. Over a 28-month follow-up, overall mortality reached 62% but exceeded 80% in those with AV block requiring pacemaker implantation [24]. These findings collectively suggest that up to 20% of patients with ATTR-CA may require a CIED and that cardiac conduction abnormalities are strongly associated with increased mortality in this population.

Although studies investigating the impact of ATTR-CA related risk scores and the number of red flag findings on survival and clinical outcomes are limited, some evidence exists in the literature. In a retrospective observational study conducted in Spain, Blanco-López E. et al. reported significantly higher mortality in patients who had ≥10 red flag features in combination with elevated hs-Troponin and NT-proBNP levels [25]. Similarly, in a prospective cohort study involving 213 patients with ATTR-CA in Denmark, Sanne Bøjet L. et al. demonstrated that red flag indicators such as high-grade AV block and aortic valve disease were strongly associated with mortality and heart failure related hospitalizations. Notably, 37% of the cohort had a history of pacemaker implantation, which was independently associated with all-cause mortality [26]. Consistent with these findings, our study showed that both a total red flag count of ≥6 and an increased T-AMYLO risk score were significantly associated with increased mortality and reduced survival.

These results suggest that, in patients presenting with unexplained cardiac conduction disturbances requiring CIED implantation, both the T-AMYLO risk score and cumulative red flag burden may serve as valuable markers not only for identifying potential ATTR-CA but also for risk stratification and survival estimation.

We would like to emphasize that our study is among the first to investigate the association between ATTR-CA diagnostic scores and parameters with both all-cause mortality and primary cardiovascular outcomes specifically in patients undergoing pacemaker implantation due to cardiac conduction defects.

Although retrospective and single-center in design, our study provides novel evidence that conduction disease in patients undergoing device implantation may conceal an underlying cardiac amyloidosis phenotype. The observed association between higher T-AMYLO scores, increased red flag burden, and adverse cardiovascular outcomes underscores the importance of structured clinical evaluation rather than indiscriminate screening. While higher T-AMYLO scores and red flag findings were associated with increased mortality, the absence of confirmed CA diagnosis precludes definitive conclusions regarding the true amyloid burden in this cohort. Nevertheless, these findings may warrant further investigation into the potential role of cardiac amyloidosis screening in selected high-risk patients. Incorporating simple clinical and echocardiographic parameters, the T-AMYLO score may serve as a practical tool to improve diagnostic accuracy and enable earlier recognition of transthyretin cardiac amyloidosis in patients with unexplained conduction disease undergoing or considered for device implantation.

Several limitations of our study should be acknowledged. First, the retrospective, single-center, and observational design inherently limits the generalizability of the findings. Second, due to the study’s design, patients did not undergo confirmatory imaging such as Tc^99^-PYP bone scintigraphy or hematologic evaluations necessary for definitive amyloidosis diagnosis. Although systematic confirmation of cardiac amyloidosis was not available, the structured use of T-AMYLO and red flag parameters allowed consistent clinical risk stratification reflecting real-world scenarios. Additionally, the lack of detailed data on device type, programming, and follow-up interrogation limited the ability to control for pacing-related factors, which may have influenced long-term outcomes and the interpretation of overall pacing burden. Finally, the inclusion period and retrospective analysis overlapped with the COVID-19 pandemic, which may have adversely affected clinical outcomes and mortality during the follow-up period.

## 5. Conclusions

In this study, we demonstrated that the T-AMYLO risk score and the presence of cardiac amyloidosis associated red flag findings independently predict both all-cause mortality and primary endpoints in patients undergoing pacemaker implantation due to unexplained cardiac conduction disorders. Notably, having six or more red flag findings was strongly associated with increased mortality risk. To our knowledge, this is among the first studies to evaluate and validate the prognostic utility of the T-AMYLO score and red flag burden specifically in this patient population, offering a valuable contribution to the literature. As ATTR cardiac amyloidosis gains increasing recognition as a treatable cause of unexplained conduction disease, early identification becomes critical. Our findings highlight the importance of systematically calculating both the T-AMYLO score and total red flag burden in patients requiring device therapy for conduction abnormalities. In those deemed high risk, further diagnostic evaluation for ATTR-CA should be strongly considered. By enabling earlier diagnosis, this approach may not only clarify the underlying etiology but also improve clinical outcomes and survival through timely initiation of targeted therapies.

## Figures and Tables

**Table 1:** Clinical and Demographic Characteristics of Patients According to Primary Endpoints and All-Cause Mortality.

	Primary Endpoints (–) (*n*: 647)	Primary Endpoints (+) (*n*: 470)	*p* Value	All-Cause Mortality (–) (*n*: 761)	All-Cause Mortality (+) (*n*:346)	*p* Value
Age, (years) *	74 (24–97)	82 (36–102)	<0.001	74 (24–97)	84 (49–102)	<0.001
Male, *n* (%)	295 (46.3)	276 (58.7)	<0.001	370 (48.6)	201 (58.1)	0.003
Indications for Implantation, *n* (%) AV Blocks, *n* (%) Bundle Branch Conduction Defects (non-AV block), *n* (%) Sick Sinus Syndrome and Sinusoidal Bradycardia, *n* (%) AF with slow ventricular rate, *n* (%)	226 (35.5) 62 (9.7) 249 (39.1) 100 (15.7)	216 (46.0) 39 (8.3) 105 (22.3) 110 (23.4)	<0.001	270 (35.5) 71 (9.3) 295 (38.8) 125 (16.4)	172 (49.7) 30 (8.7) 59 (17.1) 85 (24.6)	<0.001
Types of Implanted CIEDs, *n* (%) VR pacemaker DR pacemaker VR-ICD DR-ICD CRT-D	128 (20.1) 389 (61.1) 34 (5.3) 78 (12.2) 8 (1.3)	139 (29.6) 215 (45.7) 62 (13.2) 51 (10.9) 3 (0.6)	<0.001	146 (19.2) 460 (60.4) 49 (6.4) 98 (8.6) 8 (0.7)	121 (35.0) 144 (41.6) 47 (13.6) 31 (2.8) 3 (0.3)	<0.001
Heart Failure; *n* (%) Patients with EF < 50%	111 (17.4)	163 (37.7)	<0.001	154 (20.2)	120 (34.7)	<0.001
DM, *n* (%)	216 (33.9)	221 (47.0)	<0.001	266 (35.0)	171 (49.4)	<0.001
HT, *n* (%)	499 (78.3)	423 (90.0)	<0.001	604 (79.4)	318 (91.9)	<0.001
Stroke, *n* (%)	127 (19.9)	155 (33.0)	<0.001	161 (21.2)	121 (35.0)	<0.001
CAD, *n* (%)	171 (26.8)	228 (48.5)	<0.001	250 (32.9)	149 (43.1)	0.001
CKD, *n* (%)	132 (20.7)	191 (40.6)	<0.001	159 (20.7)	164 (47.4)	<0.001
AF, *n* (%)	287 (45.1)	256 (54.5)	0.002	338 (44.4)	205 (59.2)	<0.001
Senkop-presenkop, *n* (%)	481 (75.5)	334 (71.1)	0.097	569 (74.8)	246 (71.1)	0.199
Smoker, *n* (%)	156 (24.5)	113 (24.0)	0.860	191 (25.1)	78 (22.5)	0.358
Aortic Valve Disease **, *n* (%) None Mild to Moderate Aortic Valve Disease Severe Aortic Valve Disease SAVR—TAVI	302 (47.4) 274 (43.0) 24 (3.8) 37 (5.8)	155 (33.0) 247 (52.6) 20 (4.3) 48 (10.2)	<0.001	356 (46.8) 333 (43.8) 27 (3.5) 45 (5.9)	101 (29.2) 188 (54.3) 17 (4.9) 40 (11.6)	<0.001
Medical Treatments; *n* (%) Beta Blocker Non-DHP Calcium Channel Blocker Amiodarone Class 1 Anti-Arrhythmic Drugs Digoxin ACE-i/ARB ARNI Oral Anticoagulant MRA Diuretic	462 (72.5) 96 (15.1) 52 (8.2) 27 (4.2) 114 (17.9) 491 (77.1) 30 (4.7) 439 (68.9) 187 (29.4) 491 (77.1)	391 (83.2) 81 (17.2) 80 (17.0) 17 (3.6) 116 (24.7) 420 (89.4) 20 (4.3) 368 (78.3) 221 (47.0) 445 (94.7)	<0.001 0.330 <0.001 0.610 0.006 <0.001 0.710 <0.001 <0.001 <0.001	570 (74.9) 110 (14.5) 69 (9.1) 31 (4.1) 133 (17.5) 606 (79.6) 37 (4.3) 520 (68.3) 235 (30.9) 601 (79.0)	283 (81.8) 67 (19.4) 63 (18.2) 13 (3.8) 97 (28.0) 305 (88.2) 13 (3.8) 287 (82.9) 173 (50.0) 335 (96.8)	0.011 0.039 <0.001 0.801 <0.001 <0.001 0.412 <0.001 <0.001 <0.001

* Median (max-min); ** Aortic Valve Disease is defined as mild to moderate valve disease, mild or moderate aortic regurgitation and/or stenosis, severe aortic valve disease is defined as severe aortic regurgitation and/or stenosis, and SAVR-TAVI is defined as surgical or transcatheter aortic valve replacement. ACE-i: angiotensin-converting enzyme inhibitor, ARB: angiotensin receptor blocker, AF: atrial fibrillation, ARNI: angiotensin receptor/neprilysin inhibitor, AV: atrioventricular, CRT: cardiac resynchronisation therapy, CIED: cardiac implantable electronic device, DM: diabetes mellitus, DR: atrial and ventricular 2 leads, EF: ejection fraction, HF: heart failure, HT: hypertension, ICD: implantable cardioverter-defibrillator, CAD: coronary artery disease, CKD: chronic kidney disease, MVR: mitral valve replacement, MRA: aldosterone receptor antagonist, Non-DHP: non dihydropyridine calcium channel blocker, NYHA: New York Heart Association Classification, SAVR: surgical aortic valve replacement, SVO: cerebrovascular accident, SGLT-2: sodium-glucose cotransporter-2, TAVI: transcatheter aortic valve replacement, TVR: tricuspid valve replacement, VR: ventricular single lead.

**Table 2:** Electrocardiographic and Echocardiographic Parameters of Patients According to Primary Endpoints and All-Cause Mortality.

	Primary Endpoints (–) (*n*: 647)	Primary Endpoints (+) (*n*: 470)	*p* Value	All-Cause Mortality (–) (*n*:761)	All-Cause Mortality (+) (*n*:346)	*p* Value
Rhythm, *n* (%) Patients with sinus rhythm Patients in AF rhythm Patients in pace rhythm	336 (52.7) 96 (15.1) 205 (32.2)	161 (34.3) 90 (19.1) 219 (46.6)	<0.001	396 (52.0) 120 (15.8) 245 (32.2)	101 (29.2) 66 (19.1) 179 (51.7)	<0.001
PR, msn *	200 (106–360)	200 (138–400)	0.048	200 (106–340)	210 (138–400)	0.007
QRS, msn *	130 (62–190)	140 (72–225)	<0.001	130 (62–190)	140 (72–200)	<0.001
QTc, msn *	430 (328–552)	435 (341–543)	0.025	427 (328–537)	435 (367–557)	<0.001
LBBB, *n* (%) Complete LBBB	207 (42.9)	221 (63.1)	<0.001	249 (43.2)	179 (69.6)	<0.001
RBBB, *n* (%) Complete RBBB	57 (11.8)	29 (8.3)	0.171	65 (11.3)	21 (8.2)	0.109
Fragmented QRS, *n* (%)	150 (31.2)	85 (25.4)	0.070	172 (30.1)	63 (25.2)	0.201
Mobitz Type 2 AV Block, *n* (%)	178 (27.9)	105 (22.3)	0.032	205 (26.9)	78 (22.5)	0.120
AV Complete Block, *n* (%)	181 (28.1)	180 (38.3)	<0.001	213 (28.0)	148 (42.8)	<0.001
Low Voltage **, *n* (%)	179 (28.1)	165 (35.1)	0.013	215 (28.3)	129 (37.3)	0.003
LVEF, (%) *	60 (20–65)	58 (18–65)	0.001	60 (20–65)	60 (18–65)	0.002
LA, mm *	43 (26–64)	45 (21–67)	<0.001	44 (22–65)	45 (32–66)	<0.001
LVEDd, mm *	51 (34–75)	53 (36–76)	<0.001	51 (34–75)	54 (36–76)	<0.001
IVS, mm *	11 (6–20)	12 (7–21)	0.003	11 (6–20)	12 (7–21)	0.013
PW, mm *	11 (7–17)	11 (6–20)	0.077	11 (6–17)	11 (7–20)	0.121
TAPSE, mm *	20 (10–28)	19 (11–28)	<0.001	20 (11–28)	19 (10–28)	<0.001
sPAP, mmHg *	35 (20–95)	35 (20–100)	<0.001	35 (20–90)	38 (20–100)	<0.001
LVDD, *n* (%)	444 (69.7)	356 (75.7)	0.054	535 (70.3)	257 (74.3)	0.174

* Median (maximum-minimum); ** low voltage is defined as QRS amplitude less than 10 mV for precordial leads and QRS amplitude less than 5 mV for extremity leads in standard electrocardiograms performed at 25 mm/s and 10 mV. AF atrial fibrillation, AV: atrioventricular, IVS: interventricular septum, LA: left atrium, LBBB: left bundle branch block, LVEDd: left ventricular end-diastolic diameter, LVDD: left ventricular diastolic dysfunction, LVEF: left ventricular ejection fraction, LVWT: left ventricular wall thickness, PR: PR intervale, PW: posterior wall, QRS: QRS segment duration, QTc: corrected QT interval, RBBB: right bundle branch block, sPAP: systolic pulmonary arterial pressure, TAPSE: tricuspid annular plane systolic excursion.

**Table 3:** Biochemical and Laboratory Characteristics of Patients According to Primary Endpoints and All-Cause Mortality.

	Primary Endpoints (–) (*n*: 647)	Primary Endpoints (+) (*n*: 470)	*p* Value	All-Cause Mortality (–) (*n*: 761)	All-Cause Mortality (+) (*n*: 346)	*p* Value
Hb, g/dL *	13.2 (6.1–19)	12.4 (5.2–18.3)	<0.001	13.2 (6.1–19.0)	12.2 (5.2–18.3)	<0.001
HCT, (%) *	39.1 (17.9–56.0)	37.0 (16.2–52.6)	<0.001	38.9 (17.9–56.0)	36.3 (16.2–52.6)	<0.001
PLT, 10^3^ mm^3^ *	220.0 (29.6–535.0)	204.5 (42.0–676.0)	<0.001	218.0 (29.6–535.0)	201.0 (42.0–636.0)	<0.001
WBC, 10^3^ mm^3^ *	7.2 (3.2–17.1)	7.4 (2.3–15.6)	0.220	7.1 (3.2–17.2)	7.5 (2.3–15.6)	0.033
Lymphocyte, 10^3^ mm^3^ *	1.9 (0.2–11.2)	1.5 (0.2–4.5)	<0.001	1.9 (0.2–11.2)	1.4 (0.2–4.5)	<0.001
Monocyte, 10^3^ mm^3^ *	0.57 (0.10–2.0)	0.60 (0.10–2.2)	0.010	0.56 (0.10–2.00)	0.60 (0.10–2.20)	<0.001
Neutrophil, 10^3^ mm^3^ *	4.3 (0.3–11.3)	4.7 (1.5–10.8)	<0.001	4.3 (0.3–11.4)	4.9 (1.5–10.8)	<0.001
Creatinine, mg/dL *	0.9 (0.5–5.6)	1.1 (0.7–8.8)	<0.001	0.9 (0.5–5.6)	1.2 (0.6–8.8)	<0.001
GFR, mL/min*	69 (12–128)	54 (5–120)	<0.001	68 (12–128)	48 (5–120)	<0.001
Sodium, mmol/L *	140 (126–150)	140 (121–149)	0.190	140 (126–150)	140 (121–149)	0.083
Potassium, mmol/L *	4.5 (3.3–5.9)	4.5 (3.0–6.4)	0.210	4.5 (3.3–5.9)	4.5 (3.0–6.4)	0.911
LDL, mg/dL *	112 (15–224)	109 (38–274)	0.070	110 (15–245)	112 (38–270)	0.933
Troponin T, ng/mL *	0.011 (0.001–0.288)	0.016 (0.001–0.430)	<0.001	0.011 (0.001–0.340)	0.019 (0.006–0.430)	<0.001
NT-pro BNP, pg/mL *	144.7 (7.0–12533.0)	423.0 (3.5–18877.0)	<0.001	156.0 (3.5–12556.0)	636.1 (10.5–18,877.2)	<0.001
CRP, mg/L *	3.9 (0.3–75.0)	6.7 (1.0–106.0)	<0.001	3.8 (0.1–75.2)	8.6 (0.8–106.5)	<0.001

* Median (Min-Max). CRP: C reactive protein, Hb: haemoglobin, HCT: haematocrit, WBC: leukocyte, PLT: platelet, LDL: low density lipoprotein, GFR: glomerular filtration rate, HDL: high density lipoprotein, NT-pro BNP: N-terminal protein brain natriuretic peptide.

**Table 4:** Association of T-AMYLO Risk Score and Red Flag Findings of Cardiac Amyloidosis with Primary Endpoints and All-Cause Mortality.

	Primary Endpoints (–) (*n*: 647)	Primary Endpoints (+) (*n*: 470)	*p* Value	All-Cause Mortality (–) (*n*: 761)	All-Cause Mortality (+) (*n*: 346)	*p* Value
T-AMYLO Score Risk Groups, *n* (%) * Unidentified patients Low risk patients Moderate risk patients High risk patients	352 (55.3) 182 (28.6) 73 (11.5) 30 (4.7)	221 (47.0) 108 (23.0) 102 (21.7) 39 (8.3)	<0.001	414 (54.4) 218 (28.6) 93 (12.2) 36 (4.7)	159 (46.0) 72 (20.8) 82 (23.7) 33 (9.5)	<0.001
T-AMYLO Score, *n* **	1.0 (0.0–94.1)	2.9 (0.0–99.1)	<0.001	0.2 (0.0–99.1)	4.8 (1.8–99.1)	<0.001
Aortic Valve Disease, *n* (%) ***	237 (37.2)	246 (52.3)	<0.001	287 (37.7)	196 (56.6)	<0.001
Heart Failure, (LVEF > 40%), *n* (%)	354 (55.0)	301 (64.0)	0.006	423 (55.8)	226 (65.4)	0.004
Age > 65, *n* (%)	485 (76.1)	434 (92.3)	<0.001	592 (77.8)	327 (94.5)	<0.001
CTS, *n* (%)	106 (16.6)	83 (17.7)	0.656	123 (16.2)	66 (19.1)	0.233
Peripheral Neuropathy, *n* (%)	50 (7.8)	91 (19.4)	<0.001	63 (8.3)	78 (22.5)	<0.001
Autonomic Dysfunction, *n* (%)	115 (18.1)	96 (20.4)	0.321	142 (18.7)	69 (19.9)	0.615
Presence of AV Blocks ****, *n* (%)	413 (64.8)	373 (79.4)	<0.001	495 (65.0)	291 (84.1)	<0.001
Low Voltage **with** (IVS > 12 mm), *n* (%)	86 (13.5)	86 (18.3)	0.029	107 (14.1)	65 (18.8)	0.044
Low Voltage **with** (IVS < 12 mm), *n* (%)	93 (14.6)	81 (17.2)	0.234	108 (14.2)	66 (19.1)	0.038
LVDD, *n* (%)	448 (70.3)	344 (73.2)	0.297	535 (70.3)	257 (74.3)	0.174
Pseudo Q Wave, *n* (%)	27 (4.2)	33 (7.0)	0.043	31 (4.1)	29 (8.4)	0.003
IVS > 12 mm, *n* (%)	282 (44.3)	246 (52.3)	0.008	345 (45.3)	183 (52.9)	0.020
Total Number of Red Flags **	4.5 (0–9)	5.5 (1–9)	<0.001	5 (0–9)	6 (1–9)	<0.001
Total Follow-up Time, *n* (Month) **	70 (1–114)	39 (3–114)	<0.001	70 (1–114)	31 (3–107)	<0.001

* T-AMYLO Score risk group distinction was determined by evaluating the risk percentages of the patients according to the T-AMYLO score system. In this scoring system, patients with a risk percentage below 19.9% were considered as low risk, patients with a risk percentage between 20–74.9% were considered as moderate risk, and patients with a risk percentage of 75% and above were considered as high risk. Patients to whom this scoring system can be applied are those with an IVS thickness of 12 mm and above. Due to this limiting step in the T-AMYLO scoring system, the risk group cannot be determined in patients with IVS < 12 mm. For this reason, a subgroup called ‘patients with undetermined risk group’ is specified in the risk group distinction; ** median (maximum-minimum); *** aortic valve disease was considered as a red flag finding in all patients with echocardiographic evidence of stenosis and/or regurgitation accompanied by thickening and degenerative changes in the aortic leaflets; **** the presence of AV block was considered as a red flag finding in all patients with symptomatic bradycardia in the presence of bifascicular block or trifascicular block, all high-grade AV blocks of Mobitz type 2 and above, AV complete blocks and AF with slow ventricular rate. AF: atrial fibrillation, AV: atrioventricular, CTS: carpal tunnel syndrome, IVS: interventricular septum, LVEF: left ventricular ejection fraction, LVDD: left ventricular diastolic dysfunction.

**Table 5:** Independent Predictors of All-Cause Mortality in CIED Patients: A Multivariable Cox Regression Model.

Independent Variables for All-Cause Mortality	Hazard Ratio (HR)	Lower–Upper (95% Confidence Interval (CI))	*p* Value
WBC	1.00	1.00–1.00	0.749
HT	0.92	0.66–1.27	0.618
Hb	0.96	0.90–1.02	0.198
GFR	0.98	0.97–0.99	<0.001
NT-pro BNP	1.01	1.00–1.02	0.001
Troponin T	0.50	0.40–6.20	0.579
CRP	1.01	1.01–1.02	<0.001
Aortic Valve Disease *	1.29	1.02–1.62	0.030
Heart Failure, (LVEF > 40%)	1.42	1.09–1.97	0.037
Low Voltages on ECG	1.10	0.89–1.36	0.367
LVEF	0.98	0.97–1.01	0.054
TAPSE	0.98	0.94–1.02	0.456
AF	1.21	0.96–1.52	0.098
DM	1.05	0.83–1.32	0.667
CAD	1.00	0.78–1.27	0.998
Peripheral Neuropathy	1.52	1.16–2.00	0.002
AV Blocks **	1.48	1.08–2.03	0.013
Complete LBBB	1.43	1.13–1.80	0.002
LVDD	0.95	0.73–1.23	0.716
IVS > 12 mm	1.20	0.86–1.67	0.274
T-AMYLO Risk Score ***	1.00	1.00–1.01	0.002

* Aortic valve disease was defined as aortic valve disease (+) in all patients with echocardiographic evidence of stenosis and/or regurgitation accompanied by thickening and degenerative changes in the aortic leaflets; ** the presence of AV block was defined as all patients with symptomatic bradycardia in the presence of bifascicular block or trifascicular block, all high-grade AV blocks of Mobitz type 2 and above, AV complete blocks and AF with slow ventricular response; *** T-AMYLO Risk Score is a scoring system consisting of 5 basic parameters and aims to determine the possible risk of Transthyretin type cardiac amyloidosis in % according to the values of these parameters. The parameters in this scoring system are age, gender, interventricular septum thickness, presence of low voltage criteria on electrocardiogram and presence of carpal tunnel syndrome. According to the presence or absence of these parameters, a risk determination as a percentage value is predicted by this scoring system. The T-AMYLO Score parameter, which was accepted as the independent variable above, was accepted as the number predicted by this score as a % value. AF: atrial fibrillation, AV: atrioventricular, CRP: c reactive protein, DM: diabetes mellitus, ECG: electrocardiogram, EF: ejection fraction, GFR: glomerular filtration rate, Hb: haemoglobin, HT: hypertension, IVS: interventricular septum, CAD: coronary artery disease, HF: heart failure, LBBB: left bundle branch block, LVDD: left ventricular diastolic dysfunction, LVEF: left ventricular ejection fraction, NT-pro BNP: N-terminal protein brain natriuretic peptide, sPAP: systolic pulmonary arterial pressure, TAPSE: tricuspid annular plane systolic excursion, WBC: white blood cells.

## Data Availability

The data presented in this study are available on request from the corresponding author due to the presence of personal patient information and institutional restrictions in accordance with ethics committee regulations.
